# The Quest for Eternal Youth: Hallmarks of Aging and Rejuvenating Therapeutic Strategies

**DOI:** 10.3390/biomedicines12112540

**Published:** 2024-11-07

**Authors:** Vharoon Sharma Nunkoo, Alexander Cristian, Anamaria Jurcau, Razvan Gabriel Diaconu, Maria Carolina Jurcau

**Affiliations:** 1 Neurology Ward, Emergency Clinical County Hospital Bihor, 410169 Oradea, Romania; 2 Department of Psycho-Neurosciences and Rehabilitation, University of Oradea, 410087 Oradea, Romania; 3 Faculty of Medicine and Pharmacy, University of Oradea, 410087 Oradea, Romania

**Keywords:** aging, hallmarks of aging, senolytics, senomorphics, rejuvenation, neurodegeneration

## Abstract

The impressive achievements made in the last century in extending the lifespan have led to a significant growth rate of elderly individuals in populations across the world and an exponential increase in the incidence of age-related conditions such as cardiovascular diseases, diabetes mellitus type 2, and neurodegenerative diseases. To date, geroscientists have identified 12 hallmarks of aging (genomic instability, telomere attrition, epigenetic alterations, loss of proteostasis, impaired macroautophagy, mitochondrial dysfunction, impaired nutrient sensing, cellular senescence, stem cell exhaustion, defective intercellular communication, chronic inflammation, and gut dysbiosis), intricately linked among each other, which can be targeted with senolytic or senomorphic drugs, as well as with more aggressive approaches such as cell-based therapies. To date, side effects seriously limit the use of these drugs. However, since rejuvenation is a dream of mankind, future research is expected to improve the tolerability of the available drugs and highlight novel strategies. In the meantime, the medical community, healthcare providers, and society should decide when to start these treatments and how to tailor them individually.

## 1. Introduction

Advances in medical care and creating healthier and safer environments for people to live in have increased life expectancy from around 50 years in the early 1900s to above 70 years in the present years [1], with lower figures for low-income countries and around or above 80 years in high-income countries [2]. Although a great achievement for human society, this comes at the cost of an unprecedented growth rate of individuals over the age of 65 both in size and proportion. Individuals older than 65 are estimated to exceed 1.6 billion by 2050 [3]. These demographic changes are associated with increased pressure and costs on society and healthcare systems because aging is the main risk factor for a series of diseases such as cancer, cardiovascular diseases, and especially neurodegenerative diseases [4].

For these reasons, research on the biology of aging has rapidly accelerated in the last two decades, to decipher the mechanisms of aging, the differences between healthy and pathological aging, and to find efficient methods to slow down aging and prevent or delay the onset of age-related diseases [5].

## 2. Hallmarks of Aging

In 2013, Lopez-Otin and coworkers identified nine molecular, cellular, and systemic features considered hallmarks of aging [6]. Since then, an additional three hallmarks were identified that fulfill the following criteria [7]:

- The time-dependent manifestation of alterations accompanies the aging process;
- Experimentally accentuating the hallmark accelerates the aging process;
- Therapeutic interventions on the hallmark decelerate or reverse aging.

There is a strong interdependence between the aging hallmarks, highlighting that aging is a complex process that must be conceived of as a whole. Each of these features could be a target in developing anti-aging therapies.

### 2.1. Genomic Instability

During life, a series of exogenous chemical, physical, and biological agents, as well as endogenous factors (oxidative stress, DNA replication errors, chemical reactions), lead to point mutations, translocations, deletions, single-strand and double-strand breaks in the DNA, or gene disruptions caused by the integration of viral genomes or transposons. Although living organisms have developed complex DNA repair and maintenance mechanisms, their efficiency diminishes with age, resulting in the genomic accumulation of damage [8].

Nuclear DNA accumulates a remarkable burden of mutations with time [9], alterations that are likely tolerated due to the high energetic costs for their complete repair [10]. Research has revealed that DNA repair mechanisms are enhanced in species with increased longevity [11]. One such mechanism could be the overexpression of sirtuins, with SIRT6 having been shown to improve double-strand break repair [12].

Genome instability is promoted by alterations in the nuclear architecture, with an age-related decline in lamin B1 [13], and facilitated by oxidative stress and activation of the tumor suppressor protein p53 [14].

Due to its high replicative index, lack of protective histones, and the mitochondrial generation of reactive oxygen species (ROS), mitochondrial DNA is even more susceptible to alterations that increase with age [15], being directly associated with age-related diseases [16].

### 2.2. Telomere Attrition

Telomeres are nucleotide sequences of TTAGGG and associated proteins (shelterins) located at the end of chromosomes that cap the ends of eukaryotic chromosomes, which are not completely replicated by DNA polymerases during DNA replication and are progressively lost with each cell division [17]. When telomeres reach a critical length, they no longer bind enough telomere-capping proteins and are sensed as exposed DNA ends, thus activating the DNA damage response and arresting cell proliferation by inducing the cell cycle inhibitors p21 and p16. In addition, deficiencies in shelterins, a group of proteins that block the DNA damage response, supplementally contribute to telomere uncapping [18]. Short telomeres, however, retain a sufficient number of telomere-binding proteins to inhibit DNA repair and fuel a persistent DNA damage signal that enforces a permanent DNA damage-induced proliferative arrest, thereby initiating and maintaining cellular senescence. Age, lifestyle, social factors, and genetic variants influence the rate of telomeric attrition that predicts the lifespan in animal species [19]. Activation of the DNA damage response at telomeres results in the formation of the telomere-associated DDR foci and telomere-induced DNA damage foci used as markers of cellular senescence in cultured tissues [20]. The telomerase complex, which elongates and protects telomeres, is composed of the telomerase reverse-transcriptase holoenzyme (TERT), the RNA template component (TERC), and the dyskerin (DKC1)–NOP10–NHP2–GAR1 tetrameric complex [21]. The regulation of TERT levels is maintained by the complex cooperation of numerous transcription factors acting as repressors or activators and by signaling pathways [22]. TERT expression, which is the rate-limiting catalytic subunit of telomerase, is restricted to embryos and stem cells and becomes repressed in most somatic cells. However, in cancer cells the tight control of TERT transcription is disrupted by the aberrant expression of TERT promoter activators, primarily MYC, and by nuclear factor kappa B (NF-κB) signaling that is considered the master regulator of TERT activation in cancer cells [23]. Moreover, single nucleotide mutations in the proximal promoter of the TERT gene have been shown to generate binding motifs for transcription factors causing the aberrant expression of telomerase in a wide array of cancers [22]. It is true that in animal experiments, the genetic deletion of telomerase component genes, either the TERC or the TERT component, resulted in accelerated telomere shortening, activation of the telomere DNA damage response, and cellular senescence [20], while genetical or pharmacological reactivation or systemic viral transduction of TERT has been shown to reverse or improve neurodegeneration in mice [24]. However, the approach carries the risk of malignancies [25].

### 2.3. Epigenetic Alterations

A series of identified epigenetic alterations may contribute to aging and the onset of age-related diseases such as metabolic syndrome, neurodegeneration, or cancer.

Age leads to the hypomethylation of specific loci, which are mainly tumor suppressor genes, on a background of global hypomethylation [26]. The DNA methylation status was used to develop epigenetic clocks to predict mortality risk [27]. However, there is no compelling evidence that changes in DNA methylation cause aging [7].

Loss and post-translational modifications of histones have also been linked to aging via transcriptional changes or impairment of cellular homeostasis [28]. Histone-modifying enzymes such as sirtuins (with deacetylase function) have been subject to active research in geroscience. Transgenic overexpression of SIRT1 improves genomic stability in aging mice [29], while the overexpression of SIRT6 extends the lifespan [30]. All these findings suggest that an impaired deacetylase activity leads to histone relaxation and exposure of DNA to damaging agents, resulting in enhanced genomic instability [31].

The effect of non-coding RNAs, mainly of microRNAs, is just beginning to be studied in aging, and they appear to be valuable potential therapeutic targets for delaying age-related conditions [32].

Overall, in aging, gene expression undergoes significant changes, with aberrant production of messenger RNAs [33], an increased speed of RNA polymerase II [34], and changes in the expression of enzymes of the Drosha, Dicer, and Argonaute families [32], leading to mitochondrial dysfunction, alterations in protein folding, or systemic inflammation [35].

### 2.4. Loss of Proteostasis

Aging and age-related neurodegenerative diseases are characterized by the accumulation of altered and misfolded proteins that form aggregates [36]. Altered or damaged, misfolded proteins are degraded in the cell via two essential mechanisms: either via the ubiquitin-proteasome system (UPS) or via lysosome-mediated autophagy [37].

Cumulative oxidative damage of proteins and slowed translation elongation “trap” chaperones and prevent them from folding healthy proteins [38]. The diminished pool of chaperones, cumulated with the reduced function of the unfolded protein response (UPR) in the endoplasmic reticulum [39] causes the overwhelming of the UPS. Research has shown that in flies and nematodes, the overexpression of proteasome subunits increases the lifespan [40], while the administration of chaperones (4-phenylbutyrate) to aged mice improves cognition [41].

For chaperone-mediated autophagy, damaged proteins first bind to heat shock protein HSP70, followed by uptake into the lysosome via a translocation complex consisting of lysosome-associated membrane protein type 2A (LAMP2A) stabilized by luminal heat shock protein 90 (HSP90) [42]. LAMP2A expression decreases with age. Its increase via transgenic expression reduces aging features and improves the survival of the cell populations [43].

### 2.5. Impaired Macroautophagy

Macroautophagy is a cellular homeostasis mechanism in which a part of the cytosol, with organelles and vesicles, is progressively isolated in a double-membrane vesicle (the autophagosome), which fuses with lysosomes (a process mediated by SNARE proteins), allowing for digestion of the content of the autophagosome by lysosomal enzymes.

Findings from research in both animals and humans have documented a decline of autophagy with age and downregulation of the expression of crucial genes for autophagy such as *ATG5*, *ATG7*, or *BECN1* [7,44]. As demonstrated by Cassidy and coworkers in a conditional *ATG5* knockdown mouse model, the inhibition of autophagy accelerated the aging process. Restoration of the autophagic process led to a reduction in systemic inflammation and slowing of aging [45]. A series of experimental findings have confirmed that the stimulation of autophagy (either with overexpression of *ATG5*, knock-in mutations of *BECN1*, or pharmaceutical compounds that promote autophagy) increases the lifespan in mice [7], although the compound had no clear effect on aging-related features such as gait disturbance, motor incoordination, impaired hippocampal neurogenesis, or decreased CD4^+^ T-cell number [46]. Resveratrol, which promotes autophagic activity through the SIRT1-dependent deacetylation of autophagy proteins, has been shown to improve the metabolic profile and extend the healthspan of high-fat diet-fed mice, although its effects in human patients with type 2 diabetes mellitus were less impressive [47].

### 2.6. Mitochondrial Dysfunction

Mitochondria are the powerhouse of the cell, providing energy in the form of ATP [37]. Due to the accumulation of mitochondrial DNA damage, altered proteostasis leading to impairments of the respiratory chain enzymatic complexes, or dysfunctional mitochondrial dynamics and trafficking, the cellular bioenergetic balance is destabilized with age and the production of ROS increases, triggering apoptosis and inflammation [48,49].

However, interfering with mitochondrial function yielded conflicting results. Stimulating the mitochondrial function with L-carnitine supplementation had positive results in elderly men [50], while compromising mitochondrial function via inducing a hermetic response also increased the lifespan in *C. elegans* [51]. Elamipretide, a tetrapeptide that targets cardiolipin in the inner mitochondrial membrane and binds adenine-nucleotide translocator-1 to inhibit the mitochondrial membrane permeability [52] had promising effects on several aging phenotypes in mice and even in a clinical trial in patients with Barth syndrome [53].

### 2.7. Deregulated Nutrient Sensing

The nutrient-sensing network includes insulin-like growth factors (IGFs), their receptors, a series of intracellular signaling cascades including the phosphoinositide-3 kinase (PI3K)–Akt and the Ras–MEK–ERK pathways (Ras/Raf/mitogen-activated protein kinase–ERK kinase (MEK)–extracellular signal-regulated kinase (ERK)), and several transcription factors such as forkhead box O transcription factors (FOXOs) [7]. In response to glucose and amino acids, the mammalian target of rapamycin (mTOR) complex-1 (MTORC-1) modulates the activity of proteins and transcription factors, regulating protein synthesis, autophagy, ribosome and mitochondrial biogenesis, cellular metabolism, and proteasomal activity, stimulating anabolism in the presence of nutrients and inducing cellular defense mechanisms in conditions of nutrient shortage.

The somatotrophic axis involves the growth hormone that acts on receptors of hepatocytes to stimulate the production of IGFs. These, in turn, promote the formation of IGF receptors and activate the PI3K–Akt and MTORC-1 cascades [49]. In several models, engineered mutations of these pathways increased the lifespan and slowed down age-associated impairments [7]. In epidemiological studies, low IGF1 levels correlate with a low risk of death or cognitive impairment [54]. Dietary interventions and manipulation of dietary composition (such as the ketogenic diet) have been shown to extend the lifespan in animal experiments [55]. The beneficial effects of fasting have been attributed to the upregulation of autophagy-modulating genes during the night [56].

### 2.8. Cellular Senescence

Acute or chronic damage, such as genotoxic damage, telomere attrition, mitochondrial damage, infections, oxidative stress, nutrient imbalance, or oncogenic signaling, elicits a response leading to cellular senescence [57]. Moreover, a series of inflammatory mediators, chemokines, cytokines, or tumor growth factors can trigger secondary or paracrine senescence [58]. Senescent cells exhibit a stable proliferative arrest mediated by p53 and CDKN2A/p16 with their downstream effectors CDKN1A/p21 and RB1 (retinoblastoma-1), which inhibit cyclin-dependent kinases (CDKs) and transcriptional activators [57]. Another characteristic feature of cellular senescence is the depletion of nuclear lamin B1, leading to the formation of senescence-associated heterochromatin foci [59]. With age, senescent cells accumulate in various tissues at different rates, ranging from 2- to 20-fold when comparing young to old (>65 years) individuals [60].

Another consequence of the accumulation of senescent cells is the spread of senescent features to neighboring cells in a paracrine manner via the senescence-associated secretory phenotype (SASP), consisting of a series of pro-inflammatory cytokines and chemokines, growth factors, and metalloproteinases that result from the following [7]:

- Leakage of double-stranded DNA into the cytosol and activation of the cGAS/STING and TLR pathways;
- Increased mitochondrial production of ROS;
- Impairment of the lysosomal autophagy system and expansion of the lysosomal content, allowing the detection of senescence-associated β-galactosidase (SA-β-gal).

The SASP activates and recruits immune cells via chemokines and cytokines, suppresses the immune response through transforming growth factor β (TGF-β), remodels the extracellular matrix through the secretion of matrix metalloproteinases, triggers fibroblast activation and collagen deposition, and activates the proliferation of progenitor cells through the production of endothelial growth factor and platelet-derived growth factor [61].

Cellular senescence is a tumor suppressor mechanism that contributes to tissue repair mechanisms, the release of factors by the placenta during childbirth that promote passage of the infant through the birth canal, and cancer defense [62]. However, senescent cells should be cleared by the immune system after fulfilling their function. When immune clearance of senescent cells is delayed or impaired, the pathological consequences and promotion of age-related diseases become evident [7].

### 2.9. Stem Cell Exhaustion

Increasing age is associated with a reduced rate of tissue renewal. For example, the brain cells reactivate normally silent embryonic transcription programs in neural and oligodendrocyte progenitor cells to acquire the needed plasticity and multipotency [63]. However, stem cells and progenitor cells undergo the same age-related changes as cells without stem potential, leading to a loss of plasticity and reduced ability to repair tissue injuries with advancing age [7].

Related to the characteristics of stem cells, interesting experiments point toward the possibility of cell rejuvenation through transient reprogramming. In 2006, Takahashi and Yamanaka succeeded in creating induced pluripotent stem cells from adult cells by genetically manipulating Oct4, Sox2, Klf4, and c-Myc (OSKM) [64]. After c-Myc binds to a methylated region and opens chromatin, Oct4 and Sox2 interact with the enhancers and promoters of genes directly involved in the identity of the somatic cell and other reprogramming genes [65]. First, cells lose their differentiated phenotype by the repression of identity genes, followed by activation of pluripotency genes in a process extending over several weeks [66]. The changes include the reduction in p16, telomere elongation, and resetting of the DNA methylation clock [7]. If the reprogramming process is interrupted at an intermediate state, the cells regain their initial identity without showing DNA damage, epigenetic alterations, or changes in the transcriptome. In other words, cells are rejuvenated and do not re-establish the age-associated changes present before the reprogramming initiation [67]. The possibility of this rejuvenation approach has been proven in vitro for a series of tissues and even extended the lifespan in progeroid mice. However, this outcome was not replicated in wild-type mice [7,68]. However, it may have serious side effects [69]. Only the cyclic expression of the FOXM1 transcription factor has been shown to extend longevity in wild-type and progeroid mice via a still unexplained mechanism [70].

### 2.10. Defective Intercellular Communication

Intercellular communication is progressively altered with age, compromising the regulation of homeostasis. Neural, neuroendocrine, and hormonal signaling pathways exhibit progressive deficiencies [71], leading to chronic inflammation and a decline in immunosurveillance.

A series of blood-borne factors can act either as pro-aging or anti-aging ones. Chemokines like CCL11/eotaxin, β2-microglobulin, IL-6, TGF-β, or C1q complement factor act to induce features of aging, as shown by blood transfusions from aged to young mice [72], while young blood promotes repair capacity if transfused to old mice [73]. Multiple factors with rejuvenating properties have been identified depending on the tissue type: the chemokine CCL3/MIP-1α rejuvenates hematopoietic stem cells, and the metalloproteinase TIMP2 has similar effects on the hippocampus [74]; another cytokine, GDF11, has been shown to revitalize a series of tissues including the brain [75].

Changes in the composition of the extracellular matrix also contribute to age-related damage in numerous organs and tissues, causing mainly fibrosis [76]. In a complex interplay, extracellular matrix stiffness interferes with the function of senescent cells; these cells, in turn, produce matrix metalloproteinases and proteolytically generate damage-associated molecular patterns that activate pro-inflammatory pathways [76].

### 2.11. Chronic Inflammation

Pathological systemic or local phenotypes, such as arteriosclerosis, neuroinflammation, intervertebral disc degeneration, or osteoarthritis, cause an increase in the levels of circulating inflammatory cytokines and biomarkers, such as IL-6 or C reactive protein. These cytokines, together with a decline in the immune function and a shift of T cells toward pro-inflammatory TH1 and TH17 cells, lead to defective immune surveillance and a loss of self-tolerance and impair the maintenance and repair of biological barriers. The altered function of the barriers, in turn, promotes systemic inflammation [77,78]. Thymic involution leads to a diminished T-cell repertoire, with an inefficient immune response against novel antigens [79]. Together with the accumulation of extracellular debris due to the impaired function of phagocytes, the accumulation of infectious pathogens maintains a state of chronic inflammation with increased levels of IL-6 and IL-1β [80]. Moreover, the perturbations of the circadian rhythms may also contribute to promoting inflammatory pathways [81].

There is an intricate link between chronic inflammation and other hallmarks of aging. Genomic instability leads to the clonal expansion of myeloid cells with pro-inflammatory phenotypes implicated in cardiovascular aging [82]. The cytosolic translocation of nuclear and mitochondrial DNA stimulates the pro-inflammatory sensors, especially when combined with defective autophagy [83]. Excessive trophic signals activate the growth hormone/IGF1 and further the PI3K–Akt–MTORC-1 axis to ignite inflammation. The accumulation of senescent cells expressing the SASP further promotes inflammatory signaling [7].

Research has convincingly shown that interfering with the inflammatory pathways can reverse some aging characteristics. Sarcopenia in mice and cognition in rats is improved by etanercept via blocking TNF-α [84,85], and the molecule has also been evaluated as a potential therapeutic approach in Alzheimer’s disease [86]. Knockout of the NLRP3 inflammasome protein improves cognition and has been shown to extend the lifespan in mice [87]. Canakinumab, by blocking the production of IL-1β by caspase-1, reduces the incidence of age-related diseases such as hypertension, diabetes, or even lung cancer in patients with a history of myocardial infarction [80].

### 2.12. Gut Dysbiosis

With its essential functions in nutrient absorption, the production of vitamins and other important metabolites, as well as its signaling to the brain and other organs, the gut microbiota has provoked considerable interest in recent years as a promoter of aging and of conditions such as type 2 diabetes, neurodegenerative diseases, or cardiovascular disease [88]. The bacterial diversity of the gut microbiome is established during childhood, influenced by dietary factors, lifestyle habits, and environmental factors, remains stable during adulthood, and changes in elderly individuals, with a reduced diversity [89]. Analysis of the gut microbiota in healthy elderly individuals revealed a gradual decrease in symbiotic bacterial taxa (belonging to the *Ruminococcaceae*, *Lachnospiraceae*, and *Bacteroidaceae* families) and an enrichment in health-associated groups (*Akkermansia, Bifidobacterium*, and *Christensenellaceae*) [89]. Further analysis in centenarians showed enrichment in *Alistipes putredinis* and *Odoribacter splanchnicus*, species that can generate secondary bile acids with antimicrobial effects against *Clostridioides difficile* and *Enterococcus faecium* [90].

Fecal microbiota transplantation experiments convincingly demonstrated the involvement of gut dysbiosis in accelerating aging phenotypes. Mice with progeria have increased levels of Proteobacteria and Cyanobacteria and reduced Verrucomicrobia. Transplantation of fecal microbiota from wild-type mice enhanced their lifespan [91], an outcome replicated by administering the Verrucomicrobium *Akkermansai muciniphila* [92].

The effect of the gut microbiota is likely mediated via chronic systemic inflammatory pathways. Transferring gut microbiota from old to young germ-free mice led to increased CD4+ T-cell differentiation in the spleen and increased levels of inflammatory cytokines [93], while transplants from young mice reduced the levels of interferon γ and increased the anti-inflammatory cytokine IL-4 [94].

### 2.13. The Hallmarks of Aging Are Intricately Linked

The aging hallmarks are interdependent. The experimental attenuation or accentuation of one hallmark also affects other hallmarks [7].

DNA mutations accumulate with age across all studied species and are inversely correlated with lifespan [95]. They are likely tolerated due to the high energetic cost of the repair processes, but, similar to carcinogenesis, the mutations accelerate aging only in a permissive microenvironment created by other, non-mutagenic factors [96]. In addition, mutated DNA can result in the production of altered proteins, prone to misfolding, which impair proteostasis, can interfere with autophagy and the removal of extranuclear chromatin or cytosolic DNA, deregulate nutrient sensing (because sirtuins promote DNA repair but also respond to nutrient scarcity), contribute to mitochondrial dysfunction (via mutations of mitochondrial DNA), and promote chronic inflammation (because cytosolic DNA leakage ignites inflammation). The overexpression of SIRT1 and SIRT6 in mice has been shown to extend lifespan and decrease the incidence of malignancies in old age. However, many mechanisms may contribute to this outcome: promotion of DNA repair, increased autophagy, alleviation of oxidative stress, anti-inflammatory activities of SIRTs, or the regulation of glucose and lipid metabolism [97]. Moreover, in the context of gut dysbiosis, microbial proteins and metabolites act as mutagens, whereas mutations in intestinal cells favor dysbiosis [98]. Spermidine, generated by the catabolism of arginine in the gut [99] counteracts genomic instability [100], regulates protein translation (avoiding the impairment of proteostasis) [101], stimulates macroautophagy [102], reverses lymphocyte senescence [103], has anti-inflammatory actions [104], and prevents stem cell exhaustion in muscles [105].

**Figure 1:**
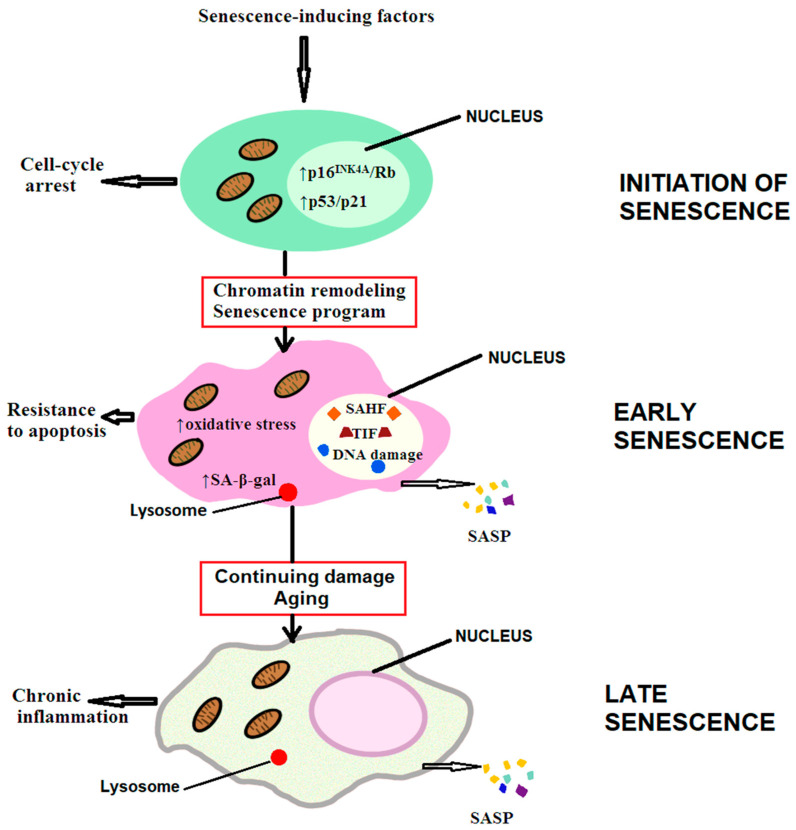
Schematically shows the progression of senescence.

The 12 hallmarks of aging described above can be classified into primary hallmarks, antagonistic hallmarks, and integrative hallmarks [7]. The primary hallmarks include damage to the genome, of telomeres, epigenetic alterations, modified proteome, and dysfunctional organelles, which accumulate in time and contribute to the aging process [106]. The antagonistic hallmarks reflect the responses to damage and include trophic signaling and anabolic reactions activated by nutrient-sensing (with beneficial roles in youth but act as pro-aging factors in the elderly), mitochondrial dysfunction (which at low levels stimulates beneficial reactions via mithormesis), and cellular senescence (which, when limited, contributes to the suppression of oncogenesis). The integrative hallmarks arise when the damage caused by the primary and antagonistic hallmarks cannot be compensated for, and they include stem cell exhaustion, alterations of intercellular communication, chronic inflammation, and gut dysbiosis [7]. The complex interplay between these hallmarks dictates the pace of aging as shown in Figure 1.

Figure 1 shows the progression of senescence. Various factors can initiate senescence via activation of the p53/p21 and p16^INK4A^/Rb pathways, leading to an irreversible cell cycle arrest. The early stages of senescence are characterized by chromatin remodeling, forming DNA segments with chromatin alterations, telomere-induced dysfunctional foci (TIF), and senescence-associated heterochromatin foci (SAHF). The senescent cells release a large repertoire of chemokines, cytokines, growth factors and enzymes, collectively known as the senescence-associated secretory phenotype (SASP), which spreads the senescent phenotype in a paracrine manner. In addition, senescent cells become resistant to apoptosis by increasing mitochondrial metabolism. Further aging and age-associated cellular damage result in chronic inflammation, a feature characteristic of late senescence.

### 2.14. Heterogeneity of Aging Across Different Tissues and Individuals

The characteristics of aging appear at different rates in the various organs and tissues. In this regard, there are two opposing views: the programmed aging rate, and the non-programmed rate [107]. The programmed aging rate theory focuses on telomere shortening, mitochondria, and oxidative stress, whereas the non-programmed aging rate theory explains the generation of damage leading to aging by individual exposure to various stressors.

However, the lifespan differs among different species by about 10^5^, suggesting that longevity is a species-specific genetically programmed trait. The lifespan of individual animals of a given species can be increased in mammals by about 1.4-fold with single gene mutations and dietary restriction [107]. Nonetheless, the expression of genes responsible for the programmed aging rate can be modified by epigenetic changes, such as the methylation and hydroxymethylation of DNA or the acetylation, methylation, phosphorylation, ubiquitination, or sumoylation of histones [108,109]. An increase in local methylation at specific promoters [110] can modulate gene expression. Similarly, changes in specific histones contribute to variations in the rate of aging by modulating gene expression through the regulation of chromatin structure [111]. Moreover, the rate of telomere shortening varies across tissues, being higher in cells with shorter mean telomere length [112]. The relative telomere length measured in a tissue sample is an average of the telomere lengths among all chromosomes within a heterogeneous population of cell types with different cell division rates and history, stem cell composition, and oxidative and inflammatory milieus, and subject to variations depending on environmental exposure and lifestyle factors [112].

Although a high burden of senescent cells can cause damage to a large area of surrounding tissue, it is unlikely that this local change would affect other organs. However, humoral factors may contribute to the spread of senescence to distant targets. A hormone secreted by brain cells triggered by the mitochondrial unfolded protein response has been shown to cause damage in the intestine in individuals with brain degenerative diseases [113]. Proteins and other substances that reach cells and the extracellular matrix in the same tissue or other organs through the circulatory system may also modulate the degree of expression of programmed aging genes in cells and tissues situated far away. The immune system could also be involved in this propagation of the senescent phenotype [114]. Both immune cells and senescent ones produce a series of pro-inflammatory factors such as IL-1 and IL-6, or TNF-α via stimulation of transcription factors like NF-κB [115]. The heterochronic blood exchange experiments, which allow the vascular connection of young and old animals and which demonstrated positive effects on the tissues of the old partner, also suggest that the senescent traits can be propagated across tissues through circulation [116].

## 3. Therapeutic Strategies Against the Features of Aging

For millennia, mankind has been searching for the fountain of youth and rejuvenating methods. Although aging affects all tissues and organs in our body, its effect on the brain is particularly devastating, because it interferes with memory, intellect, and decision-making abilities, and most profoundly alters one’s personality.

Senescence is an oncosupressive mechanism, a state of irreversible proliferative arrest triggered by the persistent activation of stress-signaling mechanisms as a safeguard for malignant transformation [117]. Senescent cells display unique metabolic alterations and develop senescent cell anti-apoptotic pathways (SCAPs), which cover Bcl-2 family members, ephrins, PI3K isoforms, p21^CIP1^, HIF-1*α*, and plasminogen-activated inhibitors 1 and 2 (PAI-1 and -2) [118]. Furthermore, senescent cells produce a wide array of pro-inflammatory cytokines, chemokines, proteases, bioactive lipids, inhibitory molecules, and other factors (the SASP) that promote the degradation of the extracellular matrix and act in a paracrine manner to induce the transition of neighboring cells into a senescent state [119].

The geroscience hypothesis states that these senescence-associated dysfunctions progress together and are crucial contributors to the pathophysiology of various diseases, loss of resilience, and age-related dysfunctions [120]. Targeting cellular senescence and other hallmarks of aging instead of individual age-associated diseases could be more cost-effective for society and healthcare. Moreover, pharmacologic depletion of senescent cells could significantly benefit the functional status of a series of age-related neurodegenerative diseases [121]. For example, whole-brain irradiation, the standard treatment for patients with brain metastases, causes progressive cognitive decline as a side effect via inducing astrocytic senescence. By selectively eliminating senescent astrocytes in irradiated transgenic mice, Yabluchanskiy and coworkers showed an improvement in the cognitive performances of irradiated animals [122]. Other researchers demonstrated that the clearance of microglia and oligodendrocyte progenitor cells expressing the senescence marker p16^INK4A^ attenuates age-related inflammation and cognitive impairment in mice [123].

The two basic classes of senotherapies are senomorphics and senolytics.

Senolytics selectively eliminate senescent cells, promoting their apoptosis by targeting critical enzymes involved in pro-survival and anti-apoptotic mechanisms, such as p53, p21, Bcl-2 family proteins, Akt, PI3K, FOXO4, and others [124]. The removal of senescent cells provides significant benefits by attenuating multiple pathological conditions, including but not limited to adipose, atherosclerosis, atrophy, cardiomyocyte hypertrophy, cataracts, renal glomerulosclerosis, sarcopenia, tumorigenesis, cancer relapse, osteoarthritis, and tau-dependent diseases [125]. Unfortunately, currently available senolytics are not selective, and targeting these anti-apoptotic mechanisms can lead to severe side effects by inducing apoptosis in non-senescent cells.

Senomorphics do not “kill” the cells, but change the phenotype of a senescent cell into a non-deleterious phenotype and thereby eliminate the SASP and the possibility of spread of the senescent phenotype by interfering with the upregulation of anti-apoptotic pathways that confer resistance to apoptosis in senescent cells [62]. Senomorphics control the SASP regulatory network including NF-*κ*B, p38MAPK, GATA4, mTOR, BRD4, and TAK1 [125]. A serious drawback is the need for continuous administration for extended periods for maximal effect.

**Figure 2:**
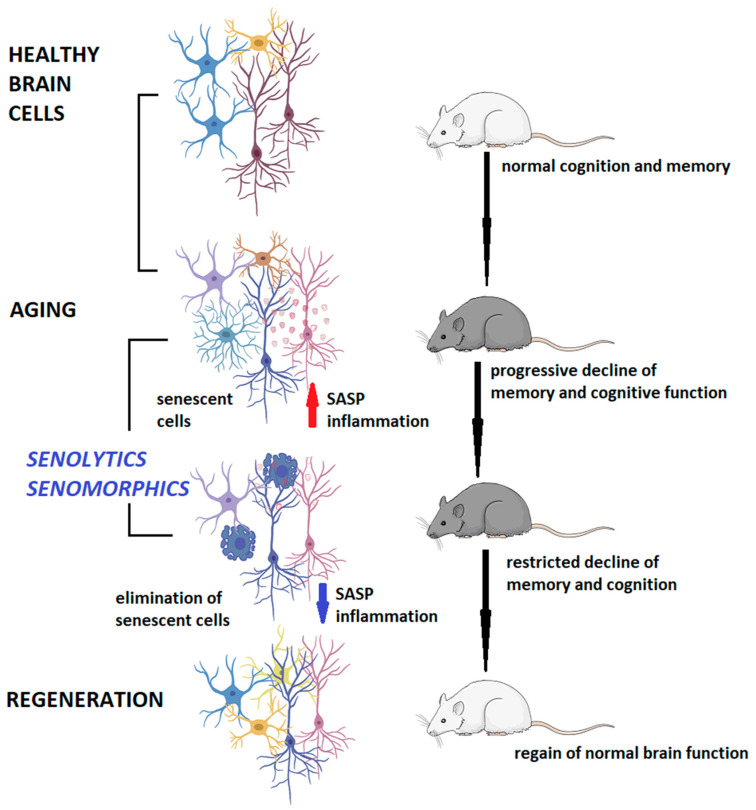
The features of the senescence of brain cells can lead to memory decline and cognitive impairment. Treating aged organisms with senolytic or senomorphic compounds either eliminates senescent cells or modulates the senescence-associated phenotype (SASP) and inflammation, reducing their deleterious effects on the brain microenvironment and allowing for the regeneration of healthy brain cells, thereby leading to the restoration of the brain functions.

Cell-based therapies are emerging, whereby senescence immune surveillance is boosted and senescent cells are cleared by T cells, macrophages, or natural killer cells [126]. Moreover, gene “editing” is rapidly advancing as a therapy for many diseases, but several aspects need to be perfected [127]. Figure 2 shows the mechanism of action of senotherapies in counteracting age-related brain dysfunction.

### 3.1. Senolytics

Senescent cells enter a persistent cell cycle arrest triggered by stress-signaling mechanisms to prevent their proliferation. However, up to 70% of senescent cells remain metabolically active and have a tissue-destructive SASP involved in spreading chronic inflammation and hallmarks of aging to other cells [128]. Alterations in macromolecules and impairments in metabolism can provide markers for the identification of senescent cells, which exhibit the following:

1. Upregulation of proteins involved in cell cycle regulation and DNA damage repair: p16^INK4A^, p21*CIP1*, p53.
2. Upregulation and excess production of SASP factors: IL-6, TNF, MMP-1, EGF.
3. Epigenetic alterations associated with DNA damage: senescence-associated heterochromatin foci, senescence-associated DNA damage foci, telomere-associated foci, phosphorylation foci of histone H2AX as markers of DNA double-strand breaks.
4. Alterations in cell morphology and nuclear membrane function associated with a reduction in lamin B1.

All the triggers known to induce senescence activate a series of anti-apoptotic and pro-survival pathways which help cancer cells to escape immune surveillance and destruction by the immune system [129,130]. As such, disabling these pathways results in the apoptosis of senescent cells, while non-senescent cells could remain viable. Because in some senescent cells, there are multiple senescent cell anti-apoptotic pathways (SCAPs), single pathway targeting is less efficient than combination approaches targeting multiple SCAPs which result in the elimination of these cells, delay the onset or alleviate senescence-associated diseases, and even extend the lifespan in model organisms [131]. Although bioinformatic analysis identified about 46 compounds that target SCAPs, only a few have escalated to preclinical trials, and only dasatinib, quercetin, and fisetin are in clinical trials for senolytic purposes [132].

Despite raising hope for preventing a variety of senescence-associated disorders, senolytic drugs, by interfering with a series of signaling pathways also used by non-senescent cells, can have serious off-target effects. To overcome this drawback, “second-generation” senolytics have been designed as galacto-conjugates of senolytics (to be decomposed by β-galactosidase and release the drug), as nanoparticles targeting cell membrane receptors of senescent cells (and taken up only by these cells), or combined with β-galactosidase-responsive coatings [133,134].

The senolytic agents can be further classified according to their mechanism of action into Bcl family inhibitors, PI3K–Akt inhibitors, FOXO regulators, cardiac glycosides, natural products and their analogs, galactose-modified senolytic drugs, proteolysis-targeting chimera (PROTAC) senolytics, and other miscellaneous compounds [135,136].

#### 3.1.1. Bcl Family Inhibitors

The Bcl family includes a series of pro-apoptotic and pro-survival proteins, such as Bcl-2, Bcl-XL, Bcl-W, or McL-1 [101]. Several compounds belonging to the Bcl family inhibitors have been developed, namely ABT263, A1331852, ABT737, and A1155463 [137].

Navitoclax (or ABT263) is an orally bioavailable molecule with a high affinity for Bcl-2, Bcl-XL, and Bcl-W by binding to the BH3 binding domain of Bcl-2 proteins and disrupting their interaction with the pro-apoptotic protein BIM [138]. In vitro, it reduced the viability of senescent human umbilical vein epithelial cells and human lung fibroblasts but did not affect human primary preadipocytes [139]. Administered orally at a dose of 50 mg/kg/day for 7 days/cycle in 2 cycles separated by a 2-week interval, it promoted senescent cell apoptosis, but with rather severe hematological side effects (thrombocytopenia and neutropenia) [140]. Phase 3 clinical trials are ongoing to evaluate its effect on myelofibrosis.

ABT737 is a precursor of navitoclax that blocks the interaction between Bcl-2 and Bcl-XL and pro-apoptotic proteins containing the BH3 domain. It eliminated senescent cells from the lungs of irradiated mice [141]. Its lack of oral bioavailability and poor solubility in water limit its potential application. A1331852 and A1155463 are Bcl-XL inhibitors with no effect on non-senescent cells and lower blood toxicity [142].

#### 3.1.2. PI3K–Akt Inhibitors

PI3K targets the serine/threonine kinase Akt, which once activated phosphorylates Bad and caspase-9, leading to their inactivation and the inhibition of apoptosis [48].

Dasatinib and quercetin are usually used together because in vitro dasatinib eliminated senescent human adipocytes but not human umbilical vein epithelial cells. The opposite was true for quercetin, while a cocktail of both compounds eliminated both types of cells [143]. Dasatinib inhibits various tyrosine kinases, thereby inhibiting cell replication and migration. It also diminishes the p21 protein levels. Quercetin, a natural flavonoid with antioxidant properties, inhibits PI3K, other kinases, and serpines [144]. The combination also inhibited Bcl-XL [145] and was successfully tested in idiopathic pulmonary fibrosis [146] and diabetic kidney disease [147]. In animal models, it increased the lifespan by about one-third [143].

#### 3.1.3. HSP90 Inhibitors

HSP90 inhibitors exert their effect by interacting with HSP90, a chaperone protein with roles in the proper folding and degradation of proteins involved in cell growth, development, and apoptosis [148]. Akt is overexpressed in senescent cells and inhibits the apoptosis of these cells by inhibiting Bax, Bad, and FOXO3A pro-apoptotic pathways. In addition, it stimulates mTOR and NF-κB signaling. Via its chaperoning machinery, HSP90 helps to maintain the structure and physiology of upregulated Akt [149]. Geldanamycin, first isolated from *Streptomyces hygroscopicus*, binds to the N-terminal ATP binding pocket of HSP90, which interferes with the ATPase chaperoning function of the protein and leads to the ubiquitin-mediated proteasomal degradation of the client proteins of HSP90. However, its senolytic function needs far higher concentrations than those required for the antitumoral effect, which, together with the severe hepatotoxicity led to the interruption of research on geldanamycin as senolytic drug [149]. Other synthetic derivatives of geldanamycin (Tanespimycin—17AAG, Alvespimycin—17-DMAG), resorcinol-containing small molecules (Ganetespib—STA9090, Onalspebib—AT13387, Luminespib—NVP-AUY922), or purine-containing HSP90 inhibitors (BIIB021, PU-H71), have been evaluated mainly as antineoplastic agents, either alone or in combination with other molecules They are only in preclinical stages of development for neurodegenerative disorders [115]. Six of these molecules have proven senolytic effects and 17-DMAG reduced age-related symptoms in mice [150].

#### 3.1.4. FOXO Regulators

Forkhead box proteins (FOXOs) are transcriptional factors that control cell growth, metabolism, and oxidative stress. FOXO4 interacts with p53 and inhibits p53-mediated apoptosis, maintaining the senescent cells viable [151]. The interaction between FOXO4 and p53 can be impaired by synthetic peptides such as FOXO4-D-retro-inverso (FOXO4-DRI) [135] or ES2, which have a higher affinity for p53 and compete with FOXO4, promoting p53-dependent apoptosis in neoplastic and senescent cells [152]. By disrupting the interaction between FOXOs and p53, these molecules allow p53 to translocate to the nucleus and trigger caspase-3/7-dependent apoptosis in senescent cells. Being composed of D-amino acids, DRI peptides are not recognized by proteases and are resistant to proteolytic degradation [153]. In animal models, FOXO4-DRI alleviated the age-related onset of hypogonadism in aged male mice by eliminating senescent Leydig cells [154].

#### 3.1.5. Other Molecules Targeting p53

Murine double minute 2 (MDM2) E3 ligase shuttles p53 from the nucleus to the cytoplasm for proteasomal degradation [155]. UBX0101 inhibits MDM2–p53 interaction and induces apoptosis in senescent cells, such as chondrocytes in a mouse model of osteoarthritis [156]. However, when tested in a phase 2 clinical trial for human osteoarthritis, it did not meet the prespecified clinical endpoints [135].

RG7112 (RO5045337) is another small molecule that inhibits MDM2–p53 interaction and has been tested in clinical trials against hematologic cancers or solid tumors. More recently, its senolytic activity was highlighted in vitro in intervertebral disc cells, where it also decreased the expression of SASP factors [157].

Targeting ubiquitin-specific peptidase 7 (USP7), an enzyme that deubiquitinates MDM2 and prevents its degradation, leads to the same result, stabilizing p53. Since all senescent cells express lower levels of p53 [158], inhibiting USP7 with small molecules such as P5091 or P22077 upregulates p53 and induces pro-apoptotic proteins (PUMA, NOXA, and FAS) [158].

#### 3.1.6. Natural Products and Their Derivatives

Fisetin is a natural flavonoid present in fruits, vegetables, and tea, with antioxidant, anti-inflammatory, antidiabetic, and neuroprotective actions, also being able to partially inhibit Bcl-2 family members such as Bcl-XL [159] and to activate caspases 7, 8, and 9 [145]. Acute or chronic administration of fisetin to wild-type old mice restored tissue homeostasis, reduced cellular senescence in many tissues, and extended lifespan [159]. A series of phase 2 studies are ongoing, evaluating the effect of fisetin in cartilage degeneration caused by osteoarthritis, on frailty in adult cancer survivors [145], in chronic kidney disease, or COVID-19 [136].

Curcumin and o-vanillin, its metabolite, have been shown to reduce SASP factors by downregulating the Nrf2 and NF-κB signaling pathways and display senolytic activity in human intervertebral disc cells [160]. Unfortunately, its poor aqueous solubility and low bioavailability limit curcumin’s clinical use. Its synthetic analog EF-24 has a more potent senolytic activity [161].

Piperlongumine is a natural alkaloid isolated from long pepper. It eliminates senescent cells via increased ROS production and inhibition of the PI3K–Akt–mTOR pathway and decreases OXR1 (oxidation resistance protein 1) [162]. A series of synthetic derivatives with the same mechanism of action have been developed and exhibit enhanced biological activity [163].

Catechins (or green tea extracts) are natural polyphenolic compounds with multiple mechanisms of action. The most abundant catechins in these extracts are epigallocatechin gallate and epigallocatechin [164]. Aside from being antioxidants and free radical scavengers, they also act on the PI3K–Akt pathway and Nrf-2 pathway and inhibit Bcl-2 expression [165].

A synthetic analog of the flavonoid wogonin, GL-V9, with anti-tumor and anti-metastatic effects in various cancer cells, has been shown to preferentially kill senescent cells in breast cancer in a ROS-dependent manner [166] by impairing mitophagy. Very likely, the tertiary amine group of GL-V9 (which is absent in wogonin) underlies the selective localization of the compound to acidic lysosomes, leading to increased oxidative stress. However, depending on the dose, GL-V9 can act either as a senolytic or as a promoter of cellular senescence, an aspect that requires further research.

#### 3.1.7. Cardiac Glycosides

A series of cardiac glycosides, such as ouabain, digoxin, or proscillardin, have been shown to eliminate senescent tumoral and primary cell lines [167]. Their senolytic activity could be ascribed to the inhibition of Na^+^/K^+^-ATPase, which causes the plasma membrane depolarization and sensitization of the senescent cells to death promoters [167]. Alternatively, they could upregulate the pro-apoptotic NOXA in senescent cells [168].

#### 3.1.8. Galactose Modified Senolytic Drugs

A common feature of senescent cells is to display increased activity of lysosomal β-galactosidase (SA-β-gal). Attaching a galactose moiety to a cytotoxic compound results in a prodrug. In senescent cells, after cellular take-up, SA-β-gal cleaves the prodrug and releases the cytotoxic compound that will eliminate the target cells [136]. A series of molecules were obtained based on this hypothesis, such as SSK1 (the chemotherapeutic gemcitabine combined with an acetyl galactose moiety and an aromatic spacer) [169], JHB75B (a carmycin analog dimer linked to two galactose groups) [170], Nav-Gal (navitoclax conjugated to an acetylated galactose group) [171], and 5FURGa (fluorouracil linked to a β-D-galactosyl moiety) [172]. Thus far, their effect has been tested only in vitro or in animal experiments.

#### 3.1.9. Proteolysis Targeting Chimera Senolytics (PROTACs)

Safer senolytics are based on the Proteolysis Targeting Chimera technology. PROTACs comprise a ligand specifically targeting a protein and an E3 ubiquitin ligase recruiting ligand connected by a linker. They lead to the ubiquitination of the target protein and use the cellular ubiquitin-proteasome system for the degradation of the targeted protein. They have prolonged activity and reduced toxicity.

PZ15227 uses ABT-263 tethered to a pomalidomide moiety, which targets it to cereblon E3 ligase ubiquitination [173], thereby leading to the proteasomal degradation of Bcl-XL. PZ15227 was proven more potent in clearing senescent cells and less toxic [173] than ABT-263.

ARV825 comprises the BET inhibitor OTX015 and the E3 ligase binder pomalidomide, promoting DNA double-strand breaks and leading to autophagy-induced apoptosis. It was shown to clear chemotherapy-induced senescent cells [174]. Considering the many proteins involved in the regulation of senescence, it is very likely that more PROTAC senolytics will be generated. However, their high molecular weight is a considerable drawback [136]. Proteins can also be targeted for degradation by using intrabodies [175].

#### 3.1.10. Other Senolytic Compounds

Other molecules with senolytic properties and diverse or yet incompletely elucidated mechanisms of action are fenofibrate [176], identified as an autophagy-promoting agent from a cell-based screening of the Prestwick Chemical Library in human senescent chondrocytes, and shown to increase PPARα expression and regulate fatty acid β-oxidation [136], or Tamatinib (R406), shown to promote apoptosis in senescent cells by inhibiting the p38MAPK and focal adhesion kinase (FAK) pathways [177,178].

Macrolide antibiotics (azithromycin and roxithromycin, but not erythromycin) were also shown to exhibit senolytic effects in human fibroblasts by inducing autophagy and driving the death of senescent cells [179].

Panobinostat is a histone deacetylase inhibitor, but it also inhibits Bcl-XL and eliminates senescent cancer cells after chemotherapy [180]. It is FDA-approved for treating refractory multiple myeloma [145] and could have potential applications as a post-chemotherapy treatment to eliminate senescent cancer cells [136].

Since senescent cells have altered bioenergetics, with impaired mitochondrial membrane potential and increased mitochondrial production of ROS [181], mitochondria-targeted tamoxifen (MitoTAM) further decreases the mitochondrial membrane potential and inhibits oxidative phosphorylation, thereby selectively eliminating senescent cells [182].

Galactose-coated nanoparticles can improve the delivery of various senolytics, being taken up preferentially by SA-β-gal-positive senescent cells [183].

#### 3.1.11. Senolytic Combination Approaches

The cell-type specificity of certain senolytics can limit their efficacy because senescent cells are heterogeneous. Combinations of senolytic drugs can increase the range of targeted senescent cellular populations via complementary or synergistic mechanisms of action. They can also be used in lower doses, reducing the cytotoxic side effects. One prominent example is dasatinib used in combination with quercetin [143]. Other evaluated combinations are piperlongumine + ABT-263, P5091 (a USP7 inhibitor) with ABT-263, or ABT-263 with tamatinib [136].

Moreover, senolytics can be combined with anticancer molecules, to increase efficiency in eliminating senescent cells and reduce drug toxicity [184]. For example, combining Taxol with Panobinostat increased efficacy against non-small cell lung cancer and head and neck squamous cell carcinoma cell lines [180]. Combined with Temozolomide, Panobinostat increased chemotherapy efficacy in glioblastoma cell lines [185].

### 3.2. Senomorphics

Senomorphics suppress the damaging effects of SASP without causing cell death.

Rapamycin (or sirolimus) is a macrolide compound isolated from Streptomyces hygroscopicus, found in Iceland. It was named after the island’s indigenous name, Rapa Nui [186]. The mTOR signaling pathway regulates cell growth, proliferation, autophagy, cellular senescence, and lifespan [187]. The mTOR protein is a component of both TORC1 and TORC2 [188]. Rapamycin reduces the phosphorylation of S6K downstream of TORC1 and inhibits mTOR signaling [187], although it may act via other mechanisms as well, such as activation of the Nrf-2 pathway or the inhibition of NF-κB signaling [117]. Its senomorphic properties have been well established in various in vitro and animal models [189]. Unfortunately, side effects such as thrombocytopenia, hyperlipidemia, metabolic dysregulation, or impaired wound healing limit its clinical use [117].

Metformin, a synthetic biguanide, exerts its senomorphic action by inhibiting the phosphorylation of IκB and inhibiting the nuclear translocation of NF-κB, the upregulation of Nrf-2-mediated glutathione peroxidase by mediating nutrient-signaling pathways via activation of AMPK and SIRT1, and the downregulation of insulin-IGF-1 signaling. Furthermore, it promotes DNA repair and augments autophagy [190]. Its ability to suppress SASP and cellular senescence has been demonstrated both in vitro and in various animal models [155]. Moreover, it has also been shown to reduce all-cause mortality and the incidence of age-related diseases in diabetic patients [191].

Resveratrol at low concentrations (below 10 μM) acts as an antioxidant and senomorphic, activates telomerase via the PI3K–Akt signaling pathway, activates SIRT1-mediated STAT3 signaling [192], inhibits NF-κB, and upregulates Nrf-2 [117], while at high concentrations (above 25 μM) it acts as a pro-oxidant and triggers senescence and apoptosis [193]. It has been shown to extend the lifespan in nematodes, flies, and fish, but in mice, its effect is obvious only in animals on a high-fat diet [194]. SIRT1 (silencing information regulator- 2-related enzyme 1) is an NAD+-dependent deacetylase that regulates a series of transcriptional pathways, the expression of which diminishes with age. Its upregulation reduces cellular senescence and extends the lifespan [195]. Synthetic sirtuin-activating compounds are currently being developed that are more potent than resveratrol and can extend the lifespan even in mice on standard diets [196].

Since NF-κB is the transcription factor that controls the expression of SASP factors and pro-inflammatory cytokines, genetic or pharmacologic inhibition of NF-κB has been shown to reduce cellular senescence both in vitro and in vivo [197].

The mitogen-activated protein kinase (MAPK) pathways deliver stress and damage signals to the nucleus. They consist of three subfamilies: the extracellular signal-regulated kinases (ERKs), the c-Jun N-terminal kinases (JNKs), and p38MAPKs. The latter are activated by stressors that also induce cellular senescence. Consequently, p38MAPK inhibitors (SB203580, UR13756, or BIRB796) were effective in blocking the SASP in cell cultures [198].

The Janus kinase–signal transducer and activator of transcription (JAK/STAT) signaling pathway also regulates SASP expression and inflammation. Inhibiting this pathway either genetically (with short inhibitory RNAs) or pharmacologically (with ruxolitinib) suppressed SASP production, reduced systemic inflammation, and reduced frailty in animal experiments [199].

Ataxia telangiectasia mutated (ATM) is a protein kinase involved in DNA damage and contributing to cellular senescence. Pharmacological inhibition of ATM with KU-55933 or KU-60019 had senomorphic effects by reducing NF-κB activation [200].

A series of natural products exhibit senomorphic properties through multiple mechanisms of action. They are mainly polyphenols (oleuropein aglycone and hydroxytyrosol in olive fruits) and flavonoids (apigenin and kaempferol) [117], but their poor bioavailability is a common concern for these compounds [201]. Table 1 summarizes the senolytic and senomorphic compounds evaluated thus far.

### 3.3. Cell-Based Therapies

Senescent cells are cleared by T cells, natural killer cells, and macrophages [208]. However, a series of mechanisms (upregulation of inhibitory ligands, shedding of stimulatory ligands, aging of the immune system) can help them to evade immune surveillance [126,209]. As such, boosting senescence immune surveillance can be an alternative to senolytic or senomorphic compounds [126].

Chimeric antigen receptor T cells (CAR-T) have an extracellular antigen binding domain targeting cell surface ligands. Binding to its target ligands elicits the cytotoxic activity of these T cells [210]. Consequently, targeting membrane-bound ligands present only on senescent cells can program CAR-T cells to kill these cells, as can CAR-NK cells [209] or natural killer cells with engineered T-cell receptors (TCRs) [126].

### 3.4. Blood-Based Rejuvenating Interventions

Administration of aged blood plasma to young mice decreases neurogenesis and promotes neuroinflammation, impairing cognitive functions [211]. Several plasmatic factors are present in the circulating blood in increasing concentrations with age and can promote aging features. They have been identified using proteomic approaches [212]. For example, the chemokine CCL11 or eotaxin-1 increases in the plasma of aged rodents and humans, can cross the blood–brain barrier (BBB) [213] and impair hippocampal neurogenesis, can activate microglia, and results in cognitive dysfunction in mice [214]. CCL2 has also been shown to contribute to a weakening of the BBB [215,216]. Components of the major histocompatibility complex I, such as β2-microglobulin, also increase with age in mice and humans [214] and decrease hippocampal neurogenesis in mice [212]. The aging hematopoietic system may supplementally drive age-associated changes in the brain [217]. The transplantation of old hematopoietic stem cells in young mice reduced synaptic density and hippocampal neurogenesis and resulted in cognitive dysfunction [218]. Mass spectrometry identified cyclophilin A as a putative agent for these alterations, although the cellular source of cyclophilin A is yet elusive [212].

The brain vascular system is a source of pro-aging factors as well. Inflammatory stimuli in the aging systemic milieu upregulate vascular cell adhesion molecule 1 (VCAM1), which promotes leukocyte interaction with the brain endothelial cells and leukocyte transmigration [211]. Neutralizing VCAM1 with antibodies or the selective genetic ablation of *Vcam1* in brain endothelial cells reduced microglial activation and improved cognition in aged mice [211]. Acid sphingomyelinase (ASM) is an enzyme produced by endothelial cells that increases with age [219] and appears to be involved in brain aging. Normalizing ASM levels via genetic approaches in mice led to increases in hippocampal synaptic spine density and improved cognition.

Risk factors or diseases such as diabetes, obesity, hypertension, or cardiovascular disease have long been linked to neurodegenerative diseases and cognitive impairment [220,221,222], as have acute inflammatory conditions such as COVID-19 with one of the mechanisms being linked to the increases in pro-inflammatory cytokines and chemokines [223].

Aging also leads to changes in the composition and functions of immune cells in the blood which, due to the weakening of the BBB and blood–CSF barriers, modifies the composition of innate and adaptive immune cells in the CNS [224]. Myeloid cells exhibit significant functional and cellular changes with age. They especially increase prostaglandin E2 signaling which promotes pro-inflammatory pathways [225]. Although the number and distribution of B cells and T cells in the cortex do not undergo significant changes with age [226], CD8+ T cells are increased in the subventricular zone of the lateral ventricles where they impair neurogenesis via increased interferon γ signaling [227]. T cells also increase in number near the sinuses, in the perivascular regions, and in the hippocampus of aged animals [228]. An increase in the population of natural killer cells was documented in the hippocampus of aged mice and humans [229].

Table 2 summarizes the pro-aging blood factors described above.

Aging phenotypes have been reversed in animal models by targeting global mechanisms through the use of heterochronic parabiosis [238]. By connecting the circulatory systems of aged and young mice, blood-borne factors generated by one individual are shared with the other. In these experiments, the young blood was reported to reverse age-related structural and molecular changes in the muscle, bone, liver, and nervous systems [239], while the young organism is affected by circulatory “pro-aging factors” [240]. Although our knowledge about the cellular targets of blood-borne factors and how they mediate rejuvenation at the systemic level remains limited, one of the most responsive cell types to young blood exposure are hematopoietic stem and progenitor cells. These are rejuvenated through the action of the proinflammatory chemokines CCL3 and CCL4 [241]. CCL3 may prevent HSPCs from over-proliferation and their differentiation ability toward T cells, which reverses the age-related myeloid bias of hematopoietic differentiation [241]. Another identified factor is osteocalcin, a bone-derived hormone that can cross the BBB, bind to neurons, and promote action potential in excitatory neurons, while also increasing BDNF levels [242]. Tissue inhibitor of metalloproteinases 2 (TIMP2) and colony-stimulating factor 2 (CSF2) are another two factors that decrease with age, cross the BBB, and enhance synaptic plasticity in the hippocampus of aged mice [74]. Although circulating levels of klotho do not change with age [243], peripheral administration of a fragment of α-klotho promotes synaptic plasticity and enhances cognition in aged mice [244], most likely by regulating inflammation at the choroid plexus blood–CSF barrier [245]. The hypothalamic gonadotropin-releasing hormone (GnRH) decreases with age due to inflammation in the aged hypothalamus. Systemic administration of GnRH enters the CNS most likely through the circumventricular organs lacking BBB and has been shown to enhance hippocampal neurogenesis in aged rodents [246]. Human serum albumin binds and transports ions, toxins, or cytokines, and exerts antioxidant effects [247]. The age-related decrease in serum albumin content has been suggested to be linked to an increased risk of atrial fibrillation [248]. The few clinical trials performed thus far were performed mainly for neurodegenerative diseases. A phase 1 clinical study showed the safety, tolerability, and feasibility of infusion of fresh frozen plasma from young donors (age 18–30) into 18 patients with AD [249], while another study proved the safety of the blood plasma-derived product GRF6019 also administered to AD patients [250]. The infusion of young blood plasma of male donors between 18 and 25 years old to Parkinson’s disease (PD) patients revealed a slight improvement in phonemic fluency together with a decrease in TNF-α [251]. A series of other clinical trials assessing the efficacy of young blood in treating frailty, cognitive decline, and even acute stroke are planned [247].

While research is ongoing, the scientific community has begun debating the ethical concerns surrounding transfusion therapies. The first question would be to what extent would donors need to be matched with the recipients, and what are the ethnic and racial disparities in access to these therapies? Given the higher HLA diversity, smaller numbers of racial and ethnic minority volunteers in donor registries, and the higher rates at which matched minority volunteers become unavailable for donation due to comorbidities in racial and ethnic minority groups, their access to these treatments would be seriously limited [252]. The costs of the procedures and the relatively high risks associated would be other prohibitive factors, mainly in poor or developing countries [253]. Another risk could be the transmission of prion diseases [254].

According to the World Health Organization reports whole blood donation exceeds 120 million units annually, in response to a massive global need [255]. This large amount needs proper storage conditions, logistics of the supply-and-demand chain, quality control, assurance parameters, and monitoring incidents during manufacturing and adverse posttransfusion reactions [256]. In addition, countries need to establish a regulatory framework for these new therapeutic strategies [257].

### 3.5. Lifestyle Approaches

Physical activity causes the release of a series of humoral factors from skeletal muscles, liver, and adipose tissue that mediate several beneficial effects. One such factor is IGF1, which crosses the BBB via receptor-mediated transport and promotes synaptic plasticity and neurogenesis in the CNS [212,258]. Selenoprotein P is a liver-derived protein found to increase after physical exercise in mice. It transports selenium across the BBB and has been shown to promote hippocampal neurogenesis [259]. Clusterin is expressed mainly by cardiomyocytes and liver cells and has been shown to increase after exercise too. It acts by inhibiting inflammation and complement activation, as well as by inhibiting apoptosis [260]. Additionally, two other exerkines, irisin and fibronectin type III domain-containing protein (FNDC5), increase after exercise [261]. Irisin is released by cleavage from FNDC5. Although the levels of irisin and FNDC5 have been found to increase in CSF from aged individuals, they are decreased in Alzheimer’s disease (AD), whereas in mouse models of AD, both peripheral and central overexpression of FNDC5 and irisin enhances synaptic plasticity, diminishes glial activation, and improves cognition [262,263].

Diet and the microbiota–gut–brain axis have been increasingly linked to neurodegenerative disorders such as AD and Parkinson’s disease (PD) [264,265,266]. The main communication between the enteric nervous system of the gastrointestinal tract and the brain is the vagus nerve [266], with almost 90% of fibers being afferent ones that reach the nucleus tractus solitarius. The vagal afferents have receptors for a series of neurotransmitters and hormones produced in the gastrointestinal tract such as serotonin, cholecystokinin, YY peptide, and ghrelin, as well as for bacterial fragments such as lipopolysaccharides. These hormones and peptides influence appetite, mood, and behavior either via the vagus nerve or by entering the systemic circulation and crossing the BBB [267]. In situations of stress, particularly if prolonged, the hypothalamic–pituitary–adrenal axis is activated, leading to the release of cortisol, but it also affects gastrointestinal motility and secretion, and changes intestinal permeability, altering the gut microbiota [268]. Conversely, the stress response can be impaired by changes in gut microbiota, even in the offspring of the manipulated animal [269]. Bacterial components such as lipopolysaccharides react with toll-like receptors leading to the production of inflammatory cytokines [270], while bacteria-derived metabolites, such as short-chain fatty acids (derived from bacterial fermentation of dietary fibers), regulate BBB integrity and act directly on the CNS [271]. Consequently, it is not surprising that the Western diet, with a high content of processed foods, saturated fat, and sugars and a low intake of fruits and vegetables, increases systemic inflammation and promotes inflammaging, which impacts the brain. The Mediterranean diet, with a high consumption of fruits, vegetables, whole grains, fiber, nuts, and olive oil and low content of sugars, saturated fat, and processed meat, improves the gut microbiota and reduces inflammation [272,273]. Research has convincingly shown that dietary interventions can preserve and even enhance cognition, executive functions, and processing speed [274,275]. The beneficial effects of nuts (antioxidant and anti-inflammatory) [276], olive oil (antioxidant, anti-inflammatory and anti-amyloidogenic) [277], cocoa flavonoids (improved neurovascular coupling, antioxidant and anti-inflammatory actions, increasing BDNF levels) [278,279], coffee (antioxidant and anti-inflammatory properties in the gut, indirectly influencing the brain via the gut–brain axis) [280], and green tea (anti-inflammatory, antioxidant, and neuroprotective) [281] are well-documented. Combining a healthy diet with physical exercise and intermittent or periodic fasting adds to the benefits [282].

Caloric restriction also has beneficial effects on reversing the hallmarks of brain senescence, possibly mediated by ketone bodies such as acetone, β-hydroxybutyrate, or acetoacetate [283].

### 3.6. An Integrated Approach to Anti-Aging Therapy

Given the intricate link between the hallmarks of aging, acting on one of these hallmarks inherently affects other hallmarks as well. For example, caloric restriction, a dietary intervention that enhances autophagy, alleviates age-related heterochromatin loss in flies [284] and activates DNA repair mechanisms [285] via sirtuins [284,286]. Physical exercise has been shown to combat telomere attrition in addition to the aforementioned anti-aging mechanisms [287]. Autophagy, as part of the quality control proteostasis programs (together with the unfolded protein response), preserves stem cell self-renewing functions and can increase stem cell expansion [288]. Transplanted mesenchymal stem cells can also modulate the autophagy of cells in injured tissues and organs, thereby contributing to their regeneration [289]. Improving proteostasis by targeting aggregated misfolded proteins (characteristically accumulated in most neurodegenerative diseases) for degradation may alleviate mitochondrial dysfunction and improve cellular bioenergetics [127,290].

The Unitary Theory of Fundamental Aging Mechanisms highlights the interdependence between the hallmarks of aging. It suggests that interventions targeting one fundamental aging hallmark may impact many or all of the others. Although exciting results derived from preclinical and early clinical trials suggest there is potential for senotherapies to alleviate age-related physical dysfunction and pathologies, more trials across different aging and disease models are needed to clearly understand the relationship between the hallmarks of aging and the individual anti-aging approaches for the benefit of the expanding population suffering from age-related health conditions [291].

Table 3 provides an overview of the hallmarks of aging, their targeted therapeutic strategies, and their current trial status.

## 4. Emerging Therapeutic Strategies

Stem cell exhaustion is an aging hallmark observed in all tissues and organs, closely linked to the accumulation of DNA damage and increased levels of p16^INK4a^ [311]. Induction of stem cell rejuvenation, reprogramming of aged hematopoietic stem cells to a pluripotent state, or inducing pluripotent stem cells (iPSCs) from aged human fibroblasts could help to restore age-associated phenotypes [312,313]. NAD+ supplementation, caloric restriction, or resveratrol-induced SIRT activation can enhance stem cell proliferation [314]. Stem cell exhaustion could also be overcome by delivering exogenous stem cells (see Table 3). While harvesting embryonic stem cells poses a series of ethical issues, mesenchymal stem cells can be derived from adipose tissue, bone marrow, umbilical cord or cord blood, and placenta [315]. Unfortunately, donor age and long-term culture negatively influence the proliferative capacity of these cells [316]. Alternatively, stem cells can be cultivated in vitro, where they release exosomes and microvesicles that contain mRNAs, microRNAs, cytosolic proteins, and lipids that modulate inflammation and can increase the proliferation rate of endogenous stem cells. Moreover, incorporating specific microRNAs into these extracellular vesicles can improve their efficacy [317].

Modulation of the gut microbiome might be beneficial in treating age-related disorders [318], as could the intake of eubiotics or probiotics (see Table 3) [319].

Non-coding RNAs control cell proliferation, differentiation, quiescence, senescence, and the cellular response to stress and immune agents [320]. Viral-mediated delivery of TERT has shown beneficial effects in animal models of aplastic anemia, idiopathic pulmonary fibrosis, or even neurodegenerative diseases [24]. However, the extra-telomeric activities of TERT pose potential risks. Research has shown that it is not telomere dysfunction per se that causes cellular senescence, but rather the pathways it engages, such as activation of the telomere DNA damage response, which depends on noncoding RNAs, such as damage-induced long non-coding RNAs and DNA damage-response RNAs. Telomeric antisense oligonucleotides (ASOs) can inhibit the activation of the DNA damage response specifically at telomeres [321] in mouse models of progeria [293]. As such, ASOs have emerged as a promising therapeutic class, with eight products already marketed or in advanced stages of clinical trials [322].

## 5. Potential Challenges in the Use of Senolytics for the Treatment of Brain Diseases

Because the survival pathways targeted by senolytics are not used only by senescent cells, the compounds used may have serious off-target effects, killing healthy cells as well. If this can be tolerated in treating lethal diseases, such as cancer, it would be unacceptable in old and frail patients already weakened by the age-related condition. At doses that are harmless for non-senescent cells, the senolytic activity is often significantly reduced. Absolute selectivity of senolytics would be possible if they could target a survival pathway used only by senescent cells; such a pathway has not been identified to date [126]. As such, caution should be exerted when translating preclinical findings into clinical trials. Ethical risk/benefit concerns should be addressed. Clinical trials should be planned at first for serious conditions, for which currently available treatments are ineffective [62]. If the side-effect profile is acceptable in these trials and the elimination of senescent cells can be demonstrated, then the use of senolytics could be tested in less serious conditions related to cellular senescence. The next step would be to address asymptomatic individuals in whom the presence of senescent cells can be demonstrated by blood, saliva, or urine tests, to delay clinically manifest disease.

The studies conducted thus far with the few currently available senolytics yielded conflicting results as to the target cell of the CNS. Depending on the model used, senolytics killed senescent neurons [323], astrocytes and microglia [122,324], or oligodendrocyte precursor cells [325]. It appears that senescent neurons are more susceptible to inhibition of the PI3K–Akt pathway, while senescent astrocytes and microglia respond better to drugs targeting Bcl-2 [126].

Moreover, neurons are post-mitotic cells that do not proliferate. When targeting senescent-like neurons, it is important to consider the consequences of removing these cells and the stage at which they can be removed without impacting overall brain functions [326]. Although adult neurogenesis does occur, it appears mainly in the hippocampal dentate gyrus and the olfactory bulb and is much more reduced in other brain areas [327].

The age-associated weakening of the BBB, partly driven by the pro-inflammatory SASP factors, creates the premises of progressive infiltration of the CNS with immune cells [328,329]. This could allow the use of cell-based senolytics such as natural killer cells or CAR-T cells. However, these are not only senolytic, but also display pro-inflammatory activities, increasing neuroinflammation and having neurotoxic side effects [126]. Nonetheless, as animal experiments have shown, senolytics can achieve their effect by acting outside the CNS and improving overall health, indirectly influencing the neurodegenerative processes. As such, drugs unable to cross the BBB could prove valuable in future research in CNS aging and neurodegeneration and allow a safer approach [126]. Overall, agents with a poor safety profile should rather be administered locally, although the paracrine spread of cellular senescence would argue against this approach.

Improved delivery methods, such as nanocarriers, could also increase the efficiency of senolytics [330]. Including navitoclax into nanoparticles engineered to selectively release the compound in the presence of SA-β-gal reduced its off-target effects [183]. Another approach would be PZ15227, a bispecific proteolysis-targeting chimera (PROTAC) that includes a binding moiety of navitoclax linked to a cereblon E3 ligase binding moiety, which kills senescent cells but exhibits less hematologic side effects because cereblon is poorly expressed in human platelets [331]. Delivering drugs via inorganic nanoparticles or included into carbon nanotubes, liposomes, or polymeric nanoparticles could also allow for lower doses and avoid systemic side effects [290].

## 6. Conclusions

Since the hallmarks of aging are strongly related, targeting several hallmarks concomitantly is likely to be more successful than targeting them individually. For example, metformin has a pleiotropic mode of action, activating the nutrient scarcity sensor AMPK, inducing autophagy, having anti-inflammatory actions, and also acting at the level of the gut microbiota [190]. A series of naturally occurring phytochemicals have pleiotropic modes of action in attenuating oxidative stress, inflammation, and the features of senescence [201], although their low bioavailability significantly limits their effect. Novel drug designs could overcome this drawback.

In previous years, we have witnessed spectacular progress in developing longevity strategies, although aging is not yet recognized as a target for drug development. However, preventing or reversing aging with drugs has been hampered by serious side effects to date. Except for lifestyle changes (healthy diet, promoting physical activity, avoidance of stress and environmental factors such as pollution, adoption of regular sleeping patterns), no drugs have yet enough evidence to support their recommendation as an anti-aging strategy. However, this could change in the future. If during the last century, we significantly expanded the lifespan, we hope that in the following century, we will be able to add quality to these years.

Nonetheless, this prospect poses a series of questions. For example, to what extent should we rely on lifestyle strategies to slow down aging, and when are more specific medical interventions needed? Targeting high-risk populations carries the risk that the interventions are programmed too late. On the other hand, the broad implementation of pharmacological, genetic, or cell-based therapies would be very costly, not affordable to poor countries or communities, and in need of complex logistics. The question of whether lifespan- and healthspan-extending treatments will need to be based on individual patient characteristics based on a metabolomic, genetic, epigenetic, or phenotypic assessment of the aging clock remains to be answered by future research in the area of geroscience and by society after a cost-effectiveness analysis.

## Figures and Tables

**Table 1:** Senolytic and senomorphic compounds.

Category	Class	Compound	Molecular Targets	Reference
Senolytics	PI3K–Akt inhibitors	Dasatinib + quercetin	PI3K–Akt pathway	[143]
	Bcl-2 inhibitors	ABT-263 (Navitoclax)	Inhibits Bcl-2, Bcl-XL, and Bcl-W	[138,202]
		ABT-737	Inhibits interaction of Bcl-2, Bcl_XL with pro-apoptotic BH3-containing proteins	[141]
		A1331852 and A1155463	Inhibit Bcl-2	[142]
	Molecules targeting p53	FOXO4-DRI	Disrupts FOXO4–p53 interaction	[154]
		UBX0101	Disrupts MDM2–p53 interaction	[156]
		RO5045337	Disrupts MDM2–p53 interaction	[157]
		P5091 or P22077	Inhibits USP7, upregulates p53	[158]
	HSP90 inhibitors	17-DMAG (alvespimycin)	Disrupts HSP90–Akt interaction, as well as the PI3K–Akt pathway	[149,150]
		17-AAG (tanespimycin)	Inhibits HSP90	[150]
		Geldanamycin	Inhibits HSP90	[150]
		Ganetespib	Inhibits HSP90	[150]
	Natural products and analogues	Fisetin	Targets Bcl-2, PI3K–AKT, p53, and NF-κB	[117,159]
		Piperlongumine and synthetic analogues	Inhibits the PI3K–Akt–mTOR pathway, decreases OXR1	[203]
		Curcumin and synthetic analog EF-24	Downregulates the Nrf2 and NF-κB pathways	[160]
		Catechins	Target the PI3K–Akt pathway and Nrf-2 pathway, and inhibits Bcl-2 expression	[165]
		GL-V9	Alkalizes lysosomes and elevates ROS levels	[166]
	Cardiac glycosides	ouabain, ouabagenin, digoxin, proscillardin, K-stropanthin	Inhibit Na^+^/K^+^-ATPase, upregulate NOXA (Bcl-2 family protein)	[168]
	Galactose-modified pro-drugs	SSK1 (gemcitabine), 5FURGa (5-fluorouracil), Nav-Gal, JHB75B (carmycin)	SA-β-gal cleaves the prodrug and releases the cytotoxic compound	[169]
	PROTACs	PZ15227	Degrades Bcl-XL	[173]
		ARV825	Promotes DNA double-strand breaks and apoptosis	[174]
	Other senolytic compounds	Panobinostat	Inhibits histone deacetylases and Bcl-XL	[180]
		Macrolide antibiotics (Azithromycin, Roxithromycin)	Induces autophagy	[179]
		Tamatinib (R406)	Inhibits p38MAPK pathway and FAK (focal adhesion kinase) pathway	[177,178]
		MitoTam	Alters mitochondrial membrane potential, inhibits oxidative phosphorylation	[182]
		Fenofibrate	PPARα agonist	[137]
Senomorphics	NF-κB inhibitors	Rapamycin	Inhibits mTOR and NF-κB pathways and activates Nrf-2 signaling	[117]
		Metformin	Inhibits NF-κB pathway and activates Nrf-2 signaling	[190]
		Resveratrol	Inhibits NF-κB and upregulates Nrf-2, Activates telomerase and activates SIRT1/STAT3 signaling	[117,192]
	p38MAPK inhibitors	SB203580, UR13756, BIRB796	Inhibits the p38MAPK pathway	[198]
	JAK/STAT inhibitors	Ruxolitinib, siRNAs	JAK1/2 inhibitor	[117]
	ATM inhibitors	KU-55933 or KU-60019	Inhibits ATM	[200]
	Statins	Atorvastatin, Pravastatin, Simvastatin, Pitavastatin	HMG-CoA reductase inhibitors	[204]
	Natural products	Oleuropein aglycone	NF-κB	[117]
		Apigenin	NF-κB	[205]
		Kaempferol	NF-κB	[206]
		Genistein	mTOR, AMPK	[207]

**Table 2:** Pro-aging blood factors.

Factor	Influence on Hallmarks of Aging	Findings in Humans	References
Blood Factors			
CCL2 (monocyte chemoattractant protein 1)	Increases vascular inflammation and BBB permeability	Increased levels are associated with impaired cognitive function in AD and MCI	[230,231,232]
CCL11 (eotaxin-1)	Decreases hippocampal neurogenesis and spine density, promotes microglial activation	Increased plasmatic concentration with age, correlates with cognitive impairment in AD and MCI	[230,232]
Β-2 microglobulin	Reduces hippocampal neurogenesis	Found increased in the plasma of aged persons and AD and HIV dementia	[214,233]
Cyclophilin A	Decreases dendritic spine density and synaptic proteins		[212,234]
VCAM1	Increases microglial activation and impairs hippocampal neurogenesis	Plasmatic levels increase with age	[211,235]
Acid sphingomyelinase	BBB disruption and decreased dendritic spine density	Plasmatic levels increase with age and in AD	[236]
Immune cells			
Peripheral myeloid cells	Increased inflammatory markers in the periphery and CNS	Expression of the prostaglandin E2 receptor 2 subtype increases with age	[125]
CD8^+^ cells	Impair neurogenesis in the subventricular zone	Increased in the subventricular zone of aged subjects	[127]
Natural killer cells	Impair hippocampal neurogenesis	Increased in the dentate gyrus of aged subjects	[237]

Abbreviations: AD—Alzheimer’s disease; BBB—blood–brain barrier; CNS—central nervous system; MCI—mild cognitive impairment; VCAM1—vascular cell adhesion molecule 1.

**Table 3:** Therapeutic strategies against the various hallmarks of aging.

Hallmark	Intervention	Species	Outcome	Clinical Trial	Outcome	Ref.
Genomic instability	SIRT6 overexpression	Mouse	Increased longevity			[30]
Telomere attrition	Telomerase activation by gene therapy	Mouse	Improvement in models of pulmonary fibrosis and aplastic anemia			[292]
	Telomeric ASOs	Hutchinson–Gilford progeria syndrome transgenic mice	Enhanced skin homeostasis and lifespan			[293]
Epi- genetic alterations	α-ketoglutarate			Phase 2 trial NCT 05706389 (ABLE)	Effect on epigenetic clock and DNA methylation	[294]
	miR-455-3p overexpression	Mouse	Extended lifespan, improved mitochondrial and cognitive functions			[295]
Loss of proteostasis	Pharmacological induction of chaperone-mediated autophagy	Mouse	Improved atherosclerosis and AD pathology in disease models			[296]
	Activation of the UPR with Guanabenz			Phase 2 trial EudraCT 2014—005367-32 in patients with ALS	Reduced the rate of progression of the disease	[297]
Disabled macroautophagy	Nordihydroguaiaretic acid	Mouse	Extended lifespan			[298]
	Nicotin- amide riboside			Phase 2 trial in patients with Parkinson’s disease NCT05589766 (N-DOSE)	Ongoing study to determine whether the compound improves proteostasis, influences histone acetylation, and decreases inflammation	[294]
Deregulated nutrient sensing	Caloric restriction	Transgenic mice	Extended lifespan by 10–30%			[299]
				Caloric restriction by 14% for 2 years—phase 2 trial NCT02695511 (CALERIE)	Improved thymopoiesis and had anti-inflammatory effects on adipose tissue	[300]
Mitochondrial dysfunction	Elamipretide(inhibits mitochondrial permeability transition)	Mouse	Improved mitochondrial function in cardiomyocytes			[301]
				Phase 2/3 clinical trial in Barth syndrome (NCT03098797, TAZPOWER)	Improved cardiac and muscle parameters and walking distance	[53]
Cellular senescence	Dasatinib + quercetin	Mouse	Increased health- and lifespan			[302]
				Phase 2 trial in chronic kidney disease (NCT02848131)—ongoing	Change in senescent cells and frailty	[294]
				Phase 2 trial in skeletal disease (NCT04313634)	Skeletal health improvement	[294]
				Phase 2 trial in AD (NCT04063124, SToMP AD)	Change in cellular senescence blood markers	[303]
				Phase 1/2 trial NCT04785300 (ALSENLITE)	Safety and tolerability of the senolytic combination	[304]
				Phase 1 trial in diabetic kidney disease (NCT02848131—ongoing)	Change in senescent cell burden	[147]
				Phase 1 trial in idiopathic pulmonary fibrosis (NCT02874989	Percentage of SASP-expressing and senescent cells	[146]
	Fisetin	Mouse	Increased healthspan and lifespan			[159]
				Phase 2 trial in frailty (NCT03430037)	Walking speed	[294]
Stem cell exhaustion	Transgenic expression of OSKM	Mouse	Reverses age-associated molecular changes			[68]
	Adenovirus-driven expression of OSK in the eye	Mouse	Restoration of visual acuity in old mice			[305]
	Allogenic MSCs			Phase 2 trial (NCT03169231)	Effect of MSCs on frailty and inflammatory biomarkers	[294]
	Autologous adipose-tissue-derived MSCs			Phase 1/2 trial NCT05827757—ongoing	Effect of MSCs on age-related low-grade inflammation	[294]
	Allogenic human MSCs delivered intravenously			Phase 1/2 trial NCT02065245 (CRATUS)	Improved immunological function, enhanced physical performance, and decreased inflammatory biomarkers	[306]
	Allogeneic cultured adult umbilical cord-derived MSCs			Phase 1 ongoing trial NCT05018767	Safety for aging frailty	[294]
	Human MSC-derived extracellular vesicles			Phase—not applicable NCT06495437—planned	Effect on age-related phenotype with impaired glucose tolerance	[294]
Altered intercellular communication	mouse	Dilution of blood from old mice with albumin/saline	Rejuvenation of multiple tissues			[307]
	Mouse	Heterochronic parabiosis	Rejuvenation in multiple tissues			[308]
Chronic inflammation	Mouse	Etanercept (blocker of TNF-α)	Increasing lifespan, preventing sarcopenia			[84]
	C57BL/6 mice	Knockout of the NLRP3 inflammasome	Improved glucose tolerance, motor performance, and cardiac aging			[87]
				Canakinumab in patients with a history of myocardial infarction—phase 3 trial (NCT01327846, CANTOS)	Reduced recurrent cardiovascular events and atherosclerotic plaque burden	[309]
Dysbiosis	Fecal microbiota transplantation from wild-type mice and *Akkermansia muciniphila* administration	Hutchinson–Gilford progeria syndrome transgenic mice	Enhanced health- and lifespan			[91]
	Microbiota transplantation from young to aged mice	Mouse	Improved immunity and brain health			[310]
	Administration of a dietary supplement with Akkermansia muciniphila and other strains			Phase 4 trial in postmenopausal women (NCT05348694, OsteoPreP)	Ongoing, will evaluate bone density, grip strength, oral glucose tolerance, and cognition	[294]

Abbreviations: AD = Alzheimer’s disease; ALS = amyotrophic lateral sclerosis; ASOs—antisense oligonucleotides; MSCs = mesenchymal stem cells; NLRP3 inflammasome = nucleotide-binding oligomerization domain, leucine rich repeat and pyrin domain containing 3 inflammasome; OSKM = Oct3/4, Sox2, Klf4, and c-Myc; TNF-α = tumor necrosis factor α; UPR = unfolded protein response.

## Data Availability

No new data were generated during the elaboration of the present manuscript.

## Abbreviations

AD	Alzheimer’s disease
Akt	protein kinase B (PKB)
AMPK	adenosine monophosphate-activated protein kinase
ASM	acid sphingomyelinase
ATM	ataxia teleangiectasia mutated
ATP	adenosine triphosphate
BBB	blood–brain barrier
BDNF	brain-derived neurotrophic factor
CAR-T	chimeric antigen receptor T cells
CDK	cyclin-dependent kinase
CNS	central nervous system
CSF	cerebrospinal fluid
cGAS	cyclic GMP-AMP synthase
DNA	deoxyribonucleic acid
EGF	epidermal growth factor
ERK	extracellular signal-regulated kinase
FAK	focal adhesion kinase
FNDC5	fibronectin type III domain-containing protein 5
FOXO	forkhead box O
GDF	growth differentiation factor
H2AX	H2A histone family member X
HIV	human immunodeficiency virus
HMG-CoA	3-hydroxy-3-methylglutaryl-coenzyme A
HSP	heat shock protein
IGF	insulin-like growth factor
IκB	inhibitor of κB
IL	interleukin
JAK	Janus kinase
JNK	c-Jun N-terminal kinase
LAMP2A	lysosome-associated membrane protein type 2A
MCI	mild cognitive impairment
MDM2	murine double minute 2
MIP	macrophage inflammatory protein
MMP	matrix metalloproteinase
mTOR	mammalian target of rapamycin
MTORC-1	mammalian target of rapamycin (mTOR) complex-1
NF-κB	nuclear factor-κB
Nrf-2	nuclear factor erythroid 2–related factor 2
OXR1	oxidation resistance protein 1
PD	Parkinson’s disease
PI3K	phosphoinositide-3 kinase
PPARα	peroxisome proliferator-activated receptor alpha
PROTAC	proteolysis-targeting chimera
RB-1	retinoblastoma-1
RNA	ribonucleic acid
ROS	reactive oxygen species
SA-β-gal	senescence-associated β-galactosidase
SASP	senescence-associated secretory phenotype
SCAPs	senescent cell anti-apoptotic pathways
SIRT	sirtuin, silencing information regulator 2-related enzyme
SNARE	soluble N-ethylmaleimide-sensitive-factor attachment receptor
STAT	signal transducer and activator of transcription
STING	stimulator of interferon genes
TERT	telomerase reverse transcriptase
TGF	transforming growth factor
TIMP	tissue inhibitor of metalloproteinases
TLR	toll-like receptor
TNF-α	tumor necrosis factor-α
UPS	ubiquitin-proteasome system
USP	ubiquitin-specific peptidase
VCAM1	vascular cell adhesion molecule 1
